# Modeling the role of gravitation in metabolic processes

**DOI:** 10.1080/19420889.2021.1914913

**Published:** 2021-07-20

**Authors:** Steve Thorne

**Keywords:** Gravity, metabolism, periodicity, chirality, circadian, circalunar, barycenter

## Abstract

All living organisms are gravitationally bound to earth’s surface and spun through three major gravitational potentials at nearly Mach 88. Along this pathway, organisms are subjected to non-isotropic strains that are repetitive in their geometry and their periodicity. Because of the relative smallness of this bias and the slow rate at which such strain accumulates, it typically goes undetected or treated stochastically as a variance from ‘best-fit’ models and woven into our empirical data. Far from being purely isotropic, equilibrium in systems co-moving with the earth possesses a dynamic component with bias defined by our orbital motion. Interestingly, biologists identify a similar bias in living organisms expressed in the chiral nature of key metabolic molecules and the periodicities of their metabolic cycles. Biologists have also identified a mean mass-specific metabolic rate that correlates well with the daily change in gravitational potential energy experienced by an organism. The evidence is only correlative, but it raises the intriguing question of whether 3 billion years of exposure to gravitational strain cycles might have led to a metabolic strategy that coupled to them. Because the subject of gravity has been omitted from most biology textbooks and, with only a few notable exceptions, relegated to the far corners of biology conferences, this paper is written with two goals in mind. The first goal is to summarize the extensive experimental record produced by biologists, botanists, and zoologists, identifying the strong correlation between metabolic processes and orbital periodicities. The second goal is to suggest experiments that might provide insight into how metabolic processes and gravitation might be so coupled.

## Introduction

In the 16^th^ Century, Nicolaus Copernicus kicked man, woman, and all other earthbound organisms out of the center of the universe and sent us on a wobbling course revolving around the sun. Unbeknownst to him at the time, he was laying down the basis for a majestic biological symphony. With time, Galileo, Kepler, and Newton each chimed in to give this symphony mathematical structure and measure. Over the subsequent three centuries, a full ensemble of physicists, mathematicians, and astronomers added harmony, melody, and overtone to this musical score. Now, biologists have joined the orchestra and brought with them the power of evolution, the nuance of diversity, and the richness and improvisation that is life. As this 21st-century curtain rises, the Copernican *‘Symphony-in-g’* is ready to be heard.

### The non-inertial motion of our orbit

**Figure 1.**
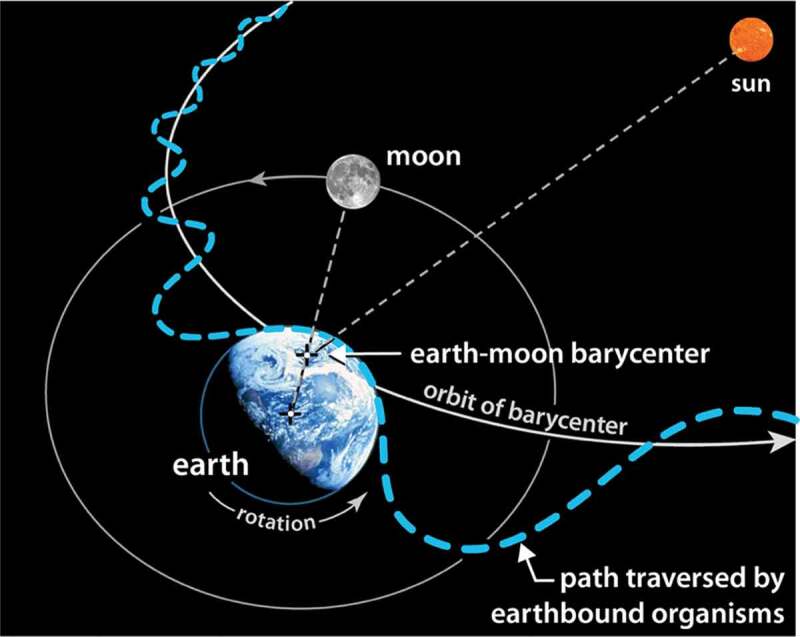
The dashed blue line illustrates the orbital path traversed by all earthbound organisms as they rotate with the earth, wobble around the earth-moon barycenter, and revolve around the sun

It is common for orbital diagrams to approximate the earth as a point mass and our orbital path as a simple circle or ellipse with the sun placed at the center or at one focus. Any organism riding aboard the earth while traversing such a simplified path would live a very uneventful inertial life. Far from the monotonous free-falling experience associated with circular or elliptical orbits – which, at best, produce seasonal variations – all earthbound organisms enjoy a much more lively existence as they are eternally twisted, shoved, pulled, and tumbled through three gravitational potentials at 88 times the speed of sound. This much more spirited path is represented schematically by the dashed blue line in Figure 1. While the scale of the path is exaggerated here, it illustrates how our true motion might be likened to riding on a roller-coaster or the teacups at Disneyland – it is filled with oscillation and rotation. As we traverse this path, we pass through a perpetually changing gravitational potential coupled to the earth, the moon, and the sun. At each moment, at all scales, our internal definition of equilibrium is being re-written.

In response to the changing equilibrium, interior elements seek new configurations and patterns of movement that will better establish coherence with the periodicity driving the system. However, equilibrium is never quite achieved, for each new iteration in configuration becomes, at best, a solution to yesterday’s problem. Such is the challenge for organisms seeking to find balance within the ever-changing orbital strain environment that fuels life.

### The experiment record, abbreviated

**Figure 2.**
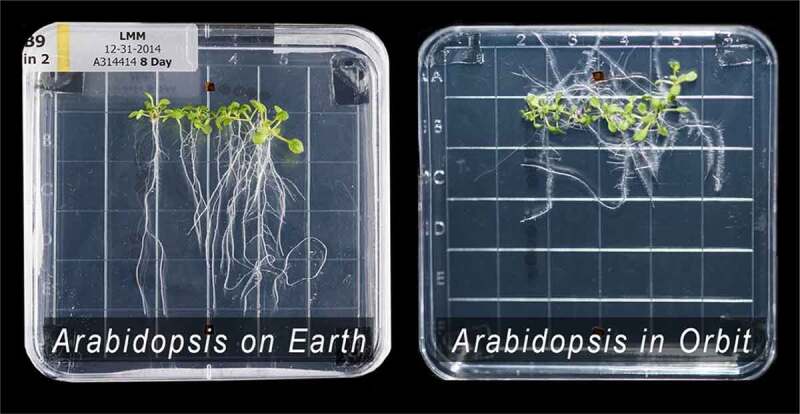
On the left: Arabidopsis after 8 days of growth on earth. On the right. Arabidopsis after 8 days of growth in orbit aboard the ISS. (Courtesy of Ferl and Paul.)

Gravity has traditionally been thought of as a static force and considered more hindrance to metabolic events than a help. When biological activity is viewed through a microscope, chemical gradients and electric potentials are seen as so powerful and dominant that it is sometimes hard to find a role for the gravitational interaction. As such, gravity has obtained the label of being an insignificant player in the biological arena. Subsequently, the subject has been omitted from most molecular biology textbooks and, with only a few notable exceptions, relegated to the far corners of biological conferences.^1^ Indeed, until NASA and the USSR started sending living organisms into space, there was little motivation for bringing biologists and physicists together and even less of a notion that their two fields might be so entwined. In 1967, two years before the first footprint was placed into the surface of the moon, a symposium was held in Sterling Forest, New York, titled *Gravity and the Organism*.^2^ It brought together botanists, biologists, zoologists, chemists, and physicists for the first time to discuss the influence gravitation might have on the properties and behavior of living matter. Their purpose: *“To explore the more interesting reactions of plants and animals to the gravitational component of their environment.”* Lectures included *‘Gravity Sensing Mechanism of the Inner Ear’* and *‘Gravimorphism in Higher Plants’*, much like the titles one finds at conferences today. After four days exploring the novelty of the problem, their collective observation was: *“It is almost strictly true that every environmental factor which varies in intensity or quality or direction – either with time or with geographical location – is exploited biologically for purposes of orientation, navigation, as an energy source or in some other fashion. **Gravitational attraction is no exception**”*. Now, five decades later, despite impressive advances in all research fronts, the full understanding of how organisms couple to that ‘*Gravitational attraction’* is still elusive. This suggests we might consider the advice offered by Richard Feynman when he said: *“It doesn’t matter how beautiful your theory is, it doesn’t matter how smart you are. If it doesn’t agree with experiment, it’s wrong.”* Many theoretical arguments can be raised to justify why gravity cannot, or should not, play a significant role in metabolic processes – but when we move away from the chalkboards, experiments tell us a different story:

There are few more concise ways to tell that story than by referencing the images in Figure 2 produced by biologists Robert Ferl and Anna-Lisa Paul from the University of Florida working in conjunction with NASA and the ISS community [1]^3^. On the left side is an image of 8-day old Arabidopsis grown in its native environment; on the right, 8-day old Arabidopsis grown in the non-native gravitational environment aboard the International Space Station (ISS). Although biologists have developed significant insights to explain how plants express gravitropic tendencies, predicting how organisms will react in new inertial environments is, at present, an empirical nightmare. If metabolic order is regulated solely by chemical reactions in combination with the thermal environment, we would not expect to see differences in the growth of Arabidopsis, for the chemical and thermal environments were precisely the same. Does this not suggest that the inertial and gravitational environment matters to metabolic order? And if so, isn’t it imperative that we establish how deep into the core of plant metabolism that coupling extends?

Below are excerpts from a 2020 summary of biological disorders observed during orbital flights over the last half-century: *Fundamental Biological Features of Spaceflight: Advancing the Field to Enable Deep-Space Exploration*.^4^ coauthored by 35 leading scientists from around the world, the paper identifies just how wide-ranging the effects are:

*“Microgravity induces cellular and molecular adaptations and changes in the genome, epigenome, as well as the proteome, and these changes create risks for a* *range of pathologies.„*

*“Exposure to microgravity induces loss of muscle mass, volume, and performance.”*
*“Spaceflight induces mitochondrial dysfunction.”*
*“Spaceflight induces a complicated pattern of immune dysfunction.”*

And, related to metabolic periodicity:

*“Circadian rhythm alterations in space have been found in a* *variety of model organisms.„*

*“Given the circadian clock system’s function in temporal coordination of nuclear, mitochondrial, cellular, and systemic processes, circadian rhythms could be a* *common denominator connecting many of the features for spaceflight biology to health risks.„*

While some of these effects are also likely due to increased radiation exposure, they clearly indicate that some form of metabolic coupling with gravitation must exist and that it somehow becomes skewed during spaceflight [2].^5^ We don’t see how one avoids the reverse conclusion that if order in metabolic processes change when organisms are subjected to the altered gravitational conditions of spaceflight, then the existing order we see in metabolic processes down here on earth must, in some way, depend on the presence of the natural earthbound gravitational component.

### About ‘microgravity’

There is a misconception about the meaning of the term *microgravity* stated most clearly by the two physicists^6^ who led off the aforementioned *‘Gravity and the Organism’* conference:

*“To say that things are weightless in a satellite leads to some misunderstanding in the way we may feel that we have removed the gravitational force. On the contrary, the gravitational force still exists, but the other forces which produce equilibrium on the surface of the earth have been removed. It is commonly [miss]-stated that objects in orbit about the earth are weightless and hence forces due to the gravitational field can no longer be detected.” ”*

Whether we adopt the Newtonian perspective that envisions objects in orbit follow trajectories that balance gravitational and centrifugal forces, or the Einsteinian view that imagines the orbit trajectory results from a balance between the momentum possessed by a mass and the local curvature of spacetime, the orbital path is best thought of as a negotiated process between two fundamentally different phenomena. Each phenomenon acts to confine the motion of the object, but one never diminishes the presence of the other in any fundamental way.

An analogy that points out the problems which arise when the term microgravity is misconstrued can be made with the way we think of air pressure. At 14 psi, the force pushing on the palm of an outstretched hand (front to back) is roughly 200 lbs. But, of course, our hand doesn’t move because there is an equal force pushing our hand in the opposite direction (back to front). We all agree that these two vectors cancel. However, that balanced state is very different from one where no pressure exists on either side of our hand. If we believed that because these vectors canceled, our hand existed in a *‘micro-pressure’* environment, this would prevent us from understanding how airplane wings generate lift and how other pressure-related forces work.

Bernard Schutz of the *Max Planck Institute for Gravitational Physics* and the *Albert Einstein Institute in Germany* points out how the equivalence principle – the basis for equating gravitational and centrifugal forces – is only approximately true and only for objects that have limited ability to measure fine-scale distortions. *“We therefore say that the equivalence principle is valid locally by not globally”*. He stresses how *‘The Real Signature of Gravity’* is locked within our interpretation of tides and tidal forces.^7^

*“For most of us the tides are romantic, primeval, poetic. Standing on an ocean beach, we might be impressed by this tangible manifestation of the gravity of the distant rock we call the Moon, but few of us would be led to reflect on how fundamental the tides are to an understanding of gravity itself. In the modern view, the real signature of gravity, the part of gravity that can’t be removed by going into free fall, is the tidal force whose most spectacular effect on Earth is to raise the ocean tides.”*

It is exciting to recognize that the scale of activity that biologists now routinely study is precisely the domain where our knowledge about gravity, tidal effects, and quantum mechanics starts to break down. Biological research that helps expose the details of how organisms can manipulate these phenomena in a controlled manner within the impossibly complex yet ordered cellular environment will likely give insight into how the uncertainty in these models is resolved. If we can’t integrate our existing models with Nature’s most subtle and pervasive force, then maybe we need to back up and revisit some of the premises we adopted when we formulated those models.

Feynman’s point, exactly.

Accordingly, without pretense for knowing exactly why any of the following correlations exist, and without claiming that any of the correlations can be or should be elevated to the status of having proved *causation*, let us simply review the experimental record related to a possible bio-gravity coupling – then we can move on to the significant experimental difficulty associated with demonstrating it.

## I: Correlations Between Metabolic Processes and Orbital Properties

### The optimum mass-specific metabolic rate: a correlation with g

**Figure 3.**
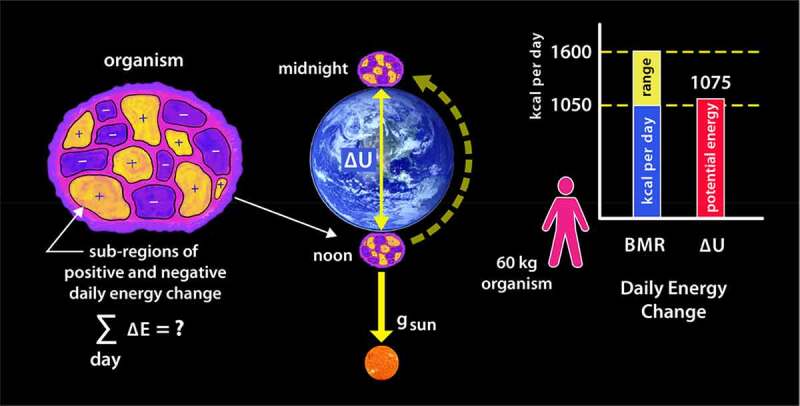
Correlation between the total daily energy consumption by an organism and the daily change in gravitational potential energy relative to the sun

While it is true that the change in gravitational potential energy *between* any two molecules, proteins, or enzymes within a cell is dwarfed by energy released through chemical reactions, it is also true that the sum of all the exergonic and endergonic reactions within an organism each day roughly equates to the daily change in the gravitational potential energy experienced by the organism. Figure 3 illustrates this interesting relationship.

If we divide an organism up into arbitrarily small regions, within which billions of high-energy chemical reactions occur each second, we expect to find localized regions of both positive and negative energy change; this is shown schematically by the yellow and blue colored regions in the left diagram. However, what is intriguing is that if we sum up each of those local energy changes over the entire body of the organism, the total daily energy change is roughly equal to the change in gravitational potential energy experienced between that organism and the sun over the same period. The middle diagram illustrates the change in position between noon and midnight relative to the sun brought about by the earth’s rotation. This rate of energy consumption is referred to as the basal metabolic rate, or BMR, and is valid when the organism is not performing extra work. The bar chart on the right illustrates the BMR and $\Delta U_{sun}$values for a 60-kg person; however, as we will see shortly, this correlation is found to be true for almost all species.

That comparison may seem odd until one realizes that when the organism rotates with the earth, its distance from the sun will vary, and therefore, work must be exerted on the mass of the organism to lift or lower it against the sun’s gravitational pull. A more detailed calculation is provided later; for now, we can simply apply the freshman-level physics approximation defining such energy change.

(1.1) $$\Delta U_{sun}\;\approx m\cdot g_{s}\cdot \left(2r_{e}\right)$$

Here m is the mass of the organism, $g_{s}$ is the gravitational acceleration toward the sun, and $2r_{e}$ is the height lifted, twice the radius of the earth. Inserting the values$\left\{m=60kg,g_{s}=5.9\times 10^{-3}m/s^{2},r_{e}=6.4\times 10^{6}m\right\}$ gives the change in the gravitational potential energy for a typical 60 kg person of $4.5\times 10^{6}joules$ (~1075 kcal) over the noon-to-midnight time interval, which falls right into the lower limit of the daily BMR for a person of this weight.^8^

**Figure 4.**
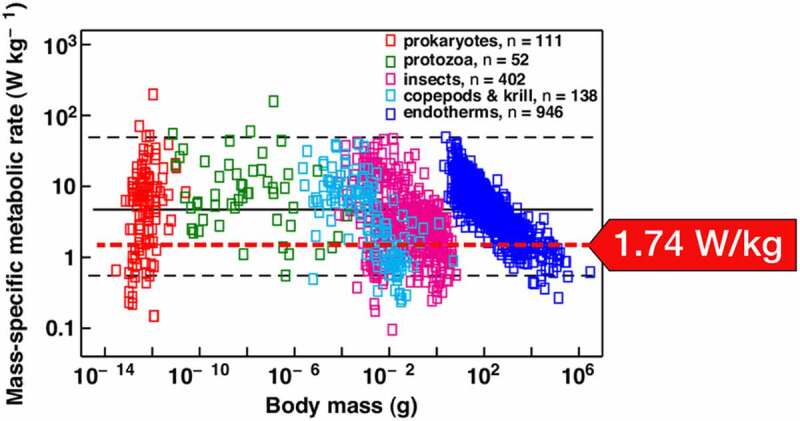
Value of the mean mass specific rate of change in the gravitational potential energy (red arrow) superimposed on the mass-specific metabolic rate observed by Makarieva et al. This range harbors organisms of practically all sizes found on earth. (Reproduced with permission from the publisher.)

This observation is made even more intriguing when combined with more general observations made by biologists about mass and metabolic rates. One study by *Makarieva et al.*,^9^ a team comprised of physicists, biologists, and botanists, investigated data on 3,006 different species, from tiny prokaryote *Francisella tularensis*, weighing in at only $10^{-17}kg$, to elephant *Elephas maximus*, weighing $4\times 10^{3}kg$. They identified how life on earth seems to function energetically with an optimum mass-specific metabolic rate – a narrow range from 0.3to9.0Watts/kg. A graphic from their paper summarizing their research is reproduced in Figure 4. Interestingly, if we generalize Eq. (1.1) above, which quantified the amplitude of the change in gravitational potential over a circadian (orbital) cycle, to a comparable mass-specific energy rate (energy per unit time per unit mass), the value is 1.74Watts/kg, which falls right into the lower limit of the power range identified by the *Makarieva* team. This value is indicated by the red arrow.

Quoting from their paper:

*“A fundamental but unanswered biological question asks how much energy, on average, Earth’s different life forms spend per unit mass per unit time to remain alive. We show that metabolism displays a* *striking degree of homeostasis across all of life. Despite the enormous biochemical, physiological, and ecological differences between the surveyed species that vary over 10^20^-fold in body mass, mean metabolic rates of major taxonomic groups displayed at physiological rest converge on a* *narrow range from 0.3 to 9 W/kg. The observed broad convergence on a* *narrow range of basal metabolic rates suggests that organismal designs that fit in this physiological window have been favored by natural selection across all of life’s major kingdoms, and that this range might therefore be considered as optimal for living matter as a* *whole.„*

The intent here is NOT to suggest that the source of metabolic energy is the change in gravitational potential energy, for over any localized region and over short timelines, chemical energy still reigns supreme. However, over the entirety of a body, over longer metabolic timelines, the systemwide change in energy seems to be approximated by the ordered change in gravitational potential energy. It is intriguing to consider whether this relationship might be a holdover from an earlier stage of our evolution. That is, prior to the development of higher energy pathways used today, such as glycolysis and the citric acid cycle, might metabolic systems have tapped the smaller strain energy generated by their wobbling motion within the gravitational field of the sun. We can make an analogy here with the role a starter motor plays in a gas vehicle. The starter motor doesn’t have the power to move the entire car around, but it gets the internal structure turning until more powerful chemical fuel can be injected. The gravitational strain energy identified here is conveniently delivered directly to the organism’s internal structure in a predictable way and on a predictable timetable. Significantly, it is still free to any organism that might have evolved a geometry and pattern of movement that was able to harvest it.

### The gravitational potential is always comprised of three interwoven components

**Figure 5.**
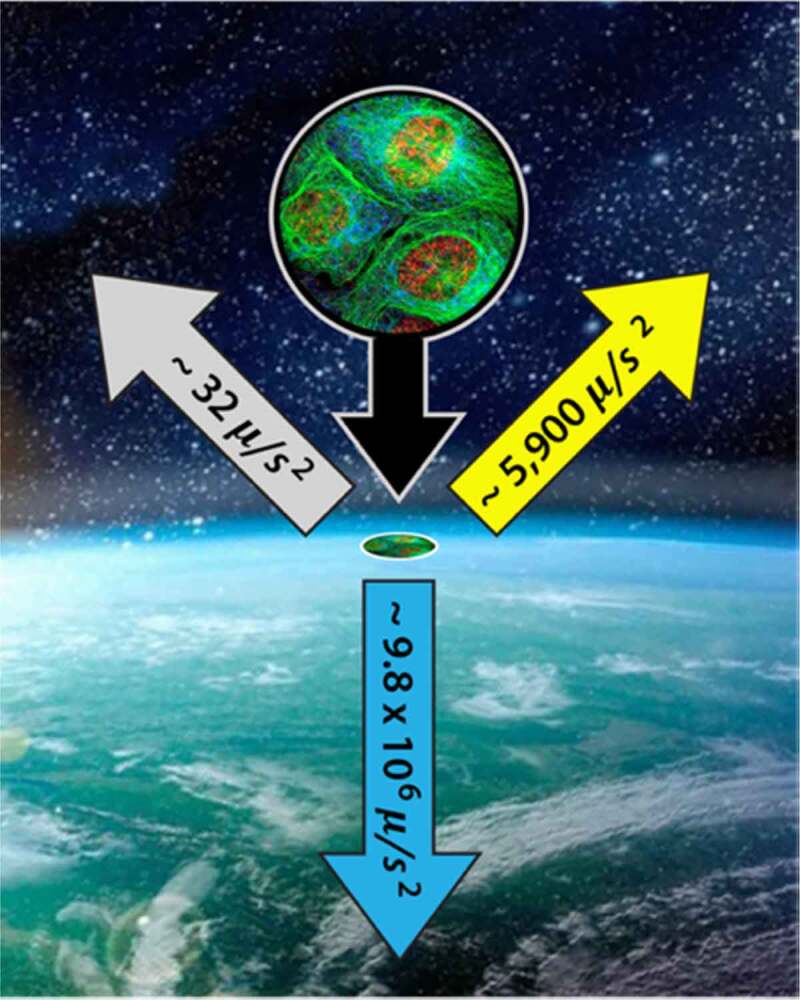
The three gravitational accelerations experienced by all earthbound organisms (values cycle with the periodicity of their respective gravitational couplings). Earth (blue arrow). Sun (yellow arrow). Moon (gray arrow)

One of the long-standing misconceptions about gravitational interactions is caused by the cavalier way in which we dismiss the gravitational fields of the sun and the moon. Contrary to statements often found in textbooks and journals, such as *“All life on Earth has evolved and adapted to a single downward vector”* [3]^10^^,^^11^ there are always three major components to the gravitational potential. They are never static, and their magnitudes and orientations change in very precise, predictable, and periodic ways.^12^ While there is nothing new or surprising about that statement, it is essential to commit to when envisioning how small organizing systems might benefit if coupled to the repetitive environmental strain patterns induced by our orbital motion. Figure 5 shows the instantaneous accelerations generated by the gradients in the gravitational fields of earth, the sun, and the moon. These accelerations are expressed in microns-per-second-squared, $\mu /s^{2}$, because their smallness appears less so when envisioning events that might be occurring within a typical cell that has a diameter of 10 microns or so. The acceleration toward the sun shifts with our orbital position from about $5700\;\;to\;\;6300\mu /s^{2}$ and oscillates by roughly $\pm 30\mu /s^{2}$ each day. Acceleration toward the moon creeps from $32\;\;to\;\;34\mu /s^{2}$ over the lunar cycle and oscillates by roughly $\pm 1\mu /s^{2}$ each day.

It is important to note that these are *not* tidal accelerations which reflect the relative acceleration between two separated point within the same gravitational field (most commonly, the two points are the center of the earth and a point on the surface). Tidal accelerations are significantly smaller and significantly more challenging to model – especially between two closely packed particles in viscoelastic fluids such as we find in a cell. Some of those difficulties are quantified later in this paper. However, even tiny acceleration differences become significant when sustained over typical metabolic cycles, which can be hours, days, or even months long.

### Circadian Periodicity and the Noon-to-Midnight Strain Cycle

**Figure 6.**
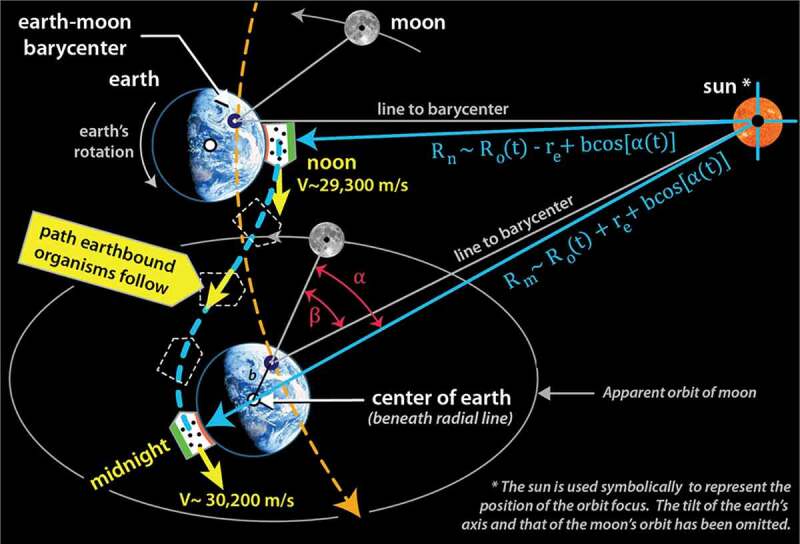
Path that organisms follow between noon and midnight (dashed blue line). Orbital path of the earth-moon barycenter indicated by dashed yellow line. Note the change in velocity and in radial distance to the sun. Note too how the offset in the barycenter and radial lines to noon and midnight

It was shown in Figure 1 how earthbound organisms traverse a wobbly, non-inertial pathway around the solar system. A more careful look at 12 hours of that pathway is shown in Figure 6; it illustrates just how rich in non-inertial character that pathway is. The ‘organism’, in this case, is represented as a container of gas molecules.

The S-shaped dashed blue line illustrates the typical *noon-to-midnight* pathway an organism follows. At noon, when the sun is directly overhead, the organism is closest to the sun, and the gravitational acceleration toward the sun is at its highest. The orbital velocity is near its minimum (29,300m/s), but over the next 12 hours, the organism will be accelerated by approximately 900 m/s; this is an increase in speed roughly four times faster than the cruising speed of a commercial airplane. Notice that during this interval, the organism is twisted and flipped relative to the sun; that motion is depicted by the orientation of the three dashed outlines. At midnight the organism will be one Earth-diameter further away from the sun, its orbital velocity will have reached its maximum (30,200m/s), and the acceleration toward the sun will be at its minimum. Significantly, the organism is not in free fall, and these accelerations result in a change in how equilibrium is defined within the organism. These patterns are reversed from midnight-to-noon, producing a natural circadian strain cycle. Since gravitational cycles oscillate in the same phase as the sunlight cycle and are not affected by weather conditions nor obscured by the physical environment, they become a good candidate for regulating metabolic processes.

We can use Figure 6 to derive the noon-to-midnight change in gravitational potential energy we summarized earlier. Choosing a 12-hour period where the angle ∝ formed by the sun, the earth, and the moon is close to zero (and thus, Cos (∝)≈1), the change can be approximated by^13^

(1.2) $$\Delta U_{noon\to midnight}\approx -\left(\frac{GM_{s}m}{R_{0}-r_{e}}\right)+\left(\frac{GM_{s}m}{R_{0}+r_{e}}\right)\approx 2mr_{e}\frac{GM_{s}}{{R_{0}}^{2}}\left(1+\frac{{r_{e}}^{2}}{{R_{0}}^{2}}+\ldots \right)\approx 2mg_{s}r_{e}$$

Inserting the mean value for the gravitational acceleration to the sun ${\overset{ˉ}{g}}_{s}=5.9\times 10^{-3}m/s^{2}$ and $r_{e}=6.4\times 10^{6}m$ for earth’s radius, dividing by the unit mass *(kg)* approximates the daily mass-specific potential energy change,^14^

(1.3) $${\hat{U}}_{n\;}=7.53\times 10^{4}J/kg$$

If we divide this value by the time interval of 12 hours (43,200 seconds), we obtain the mass-specific power obtained earlier

(1.4) $${\hat{P}}_{n\to m}=1.74Watts/kg$$

As noted above, the value in Eq. (1.4) happens to correlate with the lower range of the optimum mass-specific metabolic rate for life on earth identified by the *Makarieva* team.^15^

## The ATP correlation

If metabolic processes are coupled to periodicity in gravitational strain, then we might expect some type of correlation to show up with the value of energy provided by ATP since it represents the *unit* of metabolic energy used throughout the organism. We can calculate the work expended to raise the mass of ATP one earth diameter against the sun’s pull by using Eq. (1.2). ATP has a molar mass of 0.51kg/mol; inserting that value for m gives^16^

(1.5) $$\Delta {U_{noon\to }}_{midnight}\approx 38.4kJ/mol^{16}$$

Interestingly, this energy value falls right in the middle of the range of energy values biologists find is released during the ATP hydrolysis event, defined below in Eqs. (5a) and (5b)

(1.6a) $$ATP+H_{2}O\to ADP+P_{i}+\Delta E_{hydrolysis}$$

(1.6b) $$\Delta E_{hydrolysis}\to 28-57kJ/mol$$

If we hadn’t first identified a correlation with BMR and potential energy, we might just dismiss this ATP correlation as purely coincidental – but there exists another odd correlation with ATP that points to a possible inertial connection. Biologists observe that the total mass of ATP produced by an organism each day is roughly equal to the mass of that organism^17^; that is

(1.7) $$\frac{Mass\;of\;ATP\;produced\;per\;day}{Mass\;of\;the\;Organism}\approx 1$$

This relationship is not precise, for the ATP produced by an organism over one day also depends on the amount of work or exercise they perform. But the fact that the value is even close is intriguing since there is no apparent reason for the ATP hydrolysis energy unit to be so connected to potential energy.

**Figure 7.**
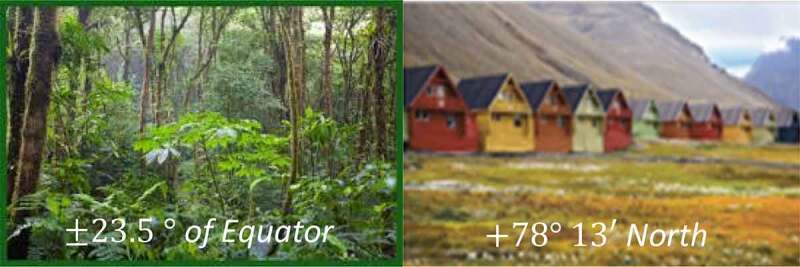
Illustration of the biodiversity gradient dependence on latitude. On the left is a picture showing the robustness of life in the tropical zone, the region within $\pm 23.5^{\circ }$ of the equator. The image on the right shows the sparseness of life at high latitudes

The periodicity of the solar gravitational cycle is invariant; however, the strain amplitude is a function of the total distance an organism is shoved back and forth in the sun’s frame each day and thus is a function of latitude. The strain defined in Eq. (1.1) was calculated for a body located at a point along the equator oscillating relative to the sun by $\left(2r_{e}\right)\;\;each\;\;day.$ At higher latitudes, the amplitude falls off roughly in proportion to Cos ($\theta_{latitude}$) . Thus, in Norway, where the latitude is $78^{\circ }\;13\prime$ N, the strain amplitude is almost 80% less than the amplitude at the equator. As Figure 7 illustrates, there is a strong latitudinal gradient in biodiversity. This is likely due to the strong latitudinal variations in temperature, sunlight, food supply, and oxygen levels. However, the $\Delta U_{sun}$ gradient could be a minor contributor. It would be interesting to investigate whether early life might have expressed a functional preference for either remaining within a similar strain environment or seeking an alternate advantageous strain environment found at adjacent latitudes.

### Seasonal Correlations

*“We are convinced that studying ‘non-circadian’ clocks contributes significantly to the full understanding of biological rhythms in general, and in particular to understanding clocks ‘in the real world … where organisms are usually confronted with more than a single temporal scale of geophysical cycles.’*

*Annual, Lunar, and Tidal Clocks*^18^

**Figure 8.**
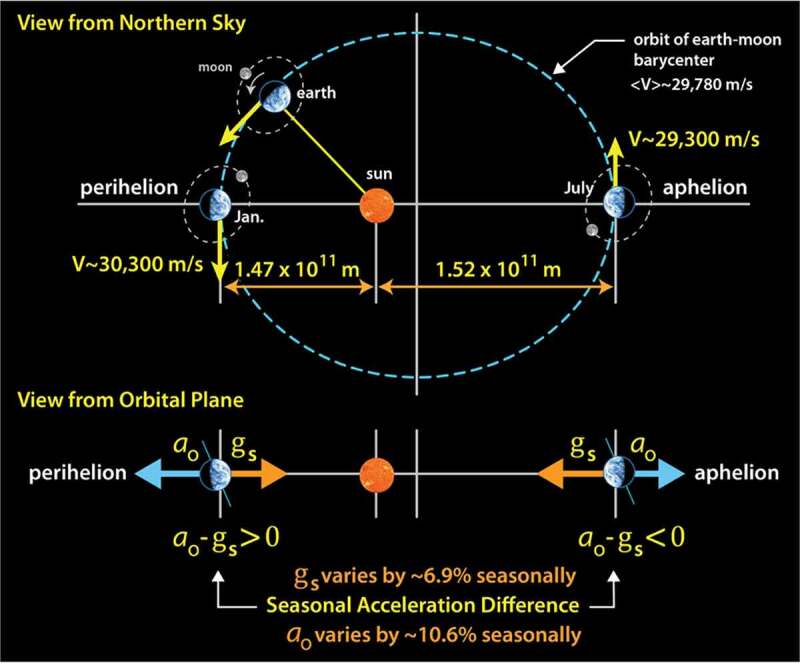
Earth’s velocity and distance from the sun vary with the seasons due to the ellipticity of our orbit. This cycles both our kinetic and potential energies, but also results in a seasonal change in the net acceleration difference between the centrifugal and gravitational accelerations

As this paper is being written, the spring season is just beginning here in Berkeley ($38^{\circ }\;latitude)$. The number of daylight hours is increasing, temperatures are starting to rise, and deciduous trees lining the bottom of campus are starting to blossom. Like most deciduous trees, they blossom roughly at the same time each year. Many environmental factors are associated with the seasons, and although temperature and sunlight duration are the easiest factors to identify and likely have the most significant influence over when the arrival of the blossoms on those trees will occur, each of our seasons also correlates with a specific strain geometry. That is, gravitation acceleration tending to pull objects toward the sun has *‘seasons’* too (the range of values was identified above). As we traverse our orbit, there is also seasonal variation in our centrifugal acceleration due to our orbit ellipticity which tends to throw us away from the sun. Interestingly, these complementary acceleration cycles never quite balance; this is shown below in Figure 8. Between July and January (aphelion and perihelion), our distance to the sun is reduced by about 3.4%, and, correspondingly, the gravitational force increases by almost 7%.^19^ Meanwhile, over that same period, the seasonal variation in the centrifugal acceleration $a_{o}$ increases by 10.6%.^20^ Those are potentially significant variations. Setting aside the numbers, we might think of July→Jan as ‘an acceleration phase’ and the Jan→July period ‘as a deceleration phase’, each with a bias defined by the geometry of our orbit. During these periods, when viewed from a spinning earth, the two complementary acceleration vectors are quietly weaving their way around each other in what might best be thought of as a seasonal dance. The strain magnitude is well under the levels that generate noticeable chemical change, and thus it may be hard to single out any particular biological event driven by the process, but it does imbue fluid equilibrium states with a fine-grained chiral bias that might be of general use to proteins.

### Lunar periodicity

**Figure 9.**
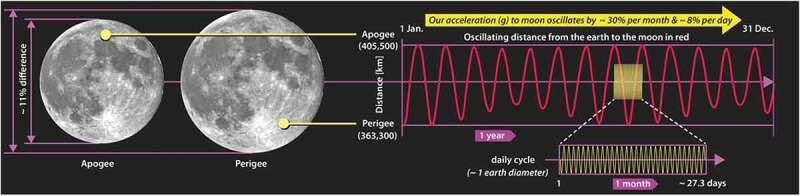
Difference in the angular size of the moon is shown on the left, and the periodicity in the moon’s distance from earth over the course of a year is shown on the right. The change in distance alters the gravitational acceleration by ~ 8% over circadian time intervals and up to 30% over a month

Perhaps the most visually engaging gravitational cycle is the lunar cycle shown in Figure 9. Due to society’s fascination with myths and movies linking the moon to weird behavior, mentioning the moon at most biological conferences would likely earn one the label of a *“lunatic”*. However, when one digs into the biological records, one finds a very robust science definitively showing that *“Circalunar and circa-semilunar rhythms are widespread among organisms.”* [4,5] Far from being a subject relegated to field observations, with the expansion of genetic analysis techniques, lunar periodicity is now studied at the level of gene expression and transcription rates. For example, in a 2019 paper by Biscontin et al. [6], which analyzed the circadian transcriptome of Antarctic krill, they report that *“About 600 genes showed a daily sinusoidal expression pattern, and the majority of these (60%) exhibited bimodal oscillatory profiles.”* In another paper from *Nature*, titled: Kaiser et al. titled *The Genomic Basis of Circadian and Circalunar Timing Adaptations of a Midge* [7] they summarize the evolutionary motivation for coupling to the moon:

*“Around new or full moon, during a* *few specific hours surrounding low tide, millions of non-biting midges of the species Clunio marinus emerge out of the sea to perform their nuptial dance. Adults live only a* *few hours, during which they mate and oviposit. They must therefore emerge synchronously and* *– given that embryonic, larval and pupal development take place in the sea* *– at a* *time when the most extreme tides reliably expose the larval habitat. The lowest low tides occur predictably during specific days of the lunar month at a* *specific time of* *day.„*

There has been no shortage of models proposed to explain this coupling. However, without gravity on their radar, almost all the modeling effort has been directed toward understanding how sunlight reflected off of the moon might regulate the cycle.^21^ Toward that end, some progress has been made identifying a possible metabolic cue associated with blue light intensity.^22^ But it is hard to imagine how moonlight alone might regulate this cycle, given the variation in cloud cover and differences in habitat that might obscure the moon’s visibility. A further modeling difficulty arose when the Kaiser group found that midges demonstrated this gene expression cycle even after they were genetically modified to prohibit the development of their optical system. It is fair to say that *“the molecular elements underpinning the detection of moonlight remain unknown.”*^23^

Then again, even a cursory look at Figure 9 suggests that the possibility of a gravitational coupling is worth investigating; the ~ 8% variation in acceleration over circadian time intervals and 30% variation over the circa-lunar (29.5-day) cycle would go a long way toward explaining the observed circalunar cycles. But since the gravitational pull from the moon is only about 1/175th as strong as the pull from the sun and the amplitude of the strain cycle proportionally smaller, the challenge to prove a gravitational coupling with circa-lunar periodicity is even more difficult.

### Periodicity in acceleration cycles aboard the ISS

In 2012, biologists Peter Barlow and Joachim Fisahn, from the School of Biological Sciences in Bristol and the Max Planck Institute in Germany, respectively, wrote a paper titled *‘Lunisolar Tidal Force and the Growth of Plant Roots, and Some Other of its Effects on Plant Movements’* [8]. They summarized a perspective gained from decades of experimental work performed by them and other colleagues identifying a possible coupling between gravity and plant metabolism. They referred to the combined gravitational force acting on organisms as the *‘lunisolar force’* and pointed to research in the previous century which contemplated a similar force; those researchers referred to it as *‘Factor X’*. Barlow and Fisahn offered the following:

*“We contend that one ever-present, but diurnally varying, environmental factor has been overlooked in leaf rhythm research, and perhaps in rhythm research in general, and that this factor fits with the properties of the mentioned ‘Factor X’: it is the diurnal variation of the combined gravitational force which the Sun and Moon exert upon the Earth.”*

This leads us into an interesting theoretical extrapolation we can extend to biological systems placed aboard the International Space Station (ISS). The station travels from west to east on an orbital inclination of 51.6 degrees, circling the earth about once every 90 minutes.^24^ Organisms relocating from the earth to the ISS have to acclimate to a new inertial environment, and their metabolic processes have a new definition of equilibrium to strive toward. Since the ISS orbit is fixed relative to the gravitational frame of the earth, and that earth’s frame is wobbling ever-so-slightly around the earth-moon barycenter, then any object seeking equilibrium on the ISS will be implementing changes that build that same wobble right into their metabolism.^25^ It is natural to wonder first, whether growing plants do indeed internalize that wobble; and second, to wonder whether that motion might be detected by experiment.

In 2015, Barlow and Fisahn teamed up with Emile Klingele, a geophysics professor from the Institute of Geodesy and Photogrammetry in Switzerland, to perform an experiment on the ISS. They tracked fine-scale leaf movements in plants placed in orbit aboard the ISS and then compared the results to cycles predicted from gravitational force calculations based on corresponding positions of the sun, the earth, and the moon. Incredibly, they found leaf movement in their plants expressed a periodicity of 45 minutes, 90 minutes, and 135 minutes.^26^ These periods are tidal periods that can be associated with the 90-minute orbital period of the ISS. It is hard to imagine how any local chemical or stochastic driven event could produce such correlations. While the biophysics behind such a coupling is far from clear, their results provide a strong argument for the conjectured connection between fine-scale metabolic processes and large-scale orbital motion.^27^

### The Chiral Bias in Our Reference Frame

**Figure 10.**
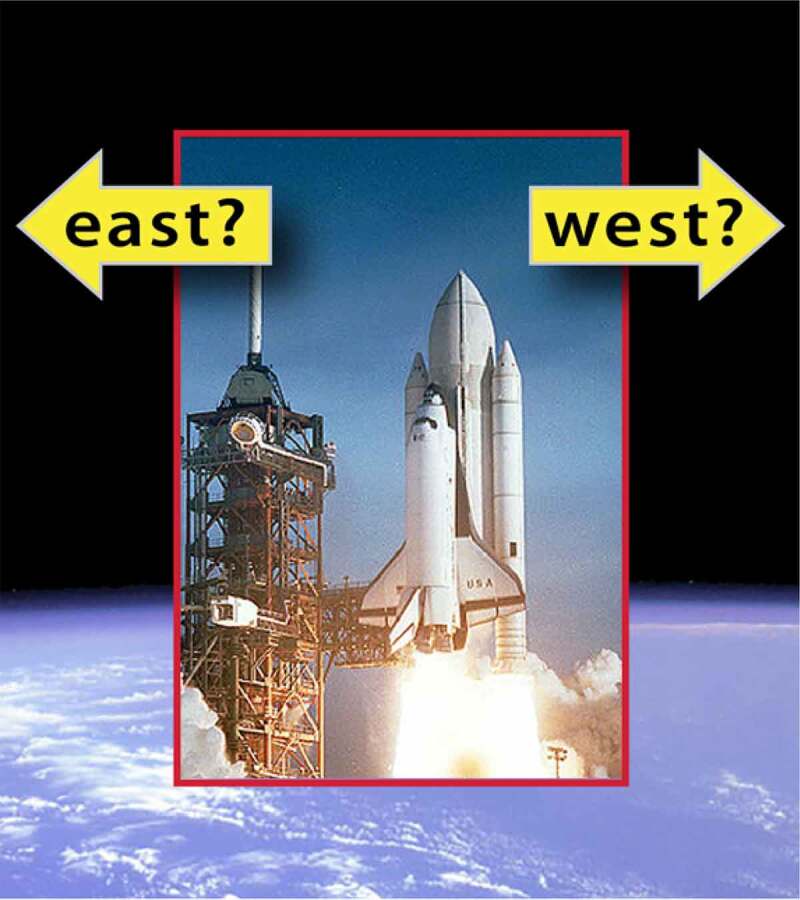
Rockets launched into orbit can take advantage of the rotational energy bias bound into their system by launching eastward in the direction of earth’s rotation

One of the first lessons taught in orbital mechanics is recognizing that objects sitting comfortably at rest on a launchpad waiting to be blasted into earth orbit or beyond are *already in orbit*. In the Copernican frame, every object in the solar system possesses a specific potential and kinetic energy. Movement always represents a *transition* between two non-inertial orbits, which typically has a geometrical bias. Movement from the earth’s surface to the earth’s orbit is a good example. While it might be difficult to imagine such an energy bias when looking through a microscope or down at one’s feet, NASA, the ESA, and other space agencies preferentially launch their rockets into eastbound directed orbits because they can capture the kinetic energy wound into the system by the earth’s rotation (Figure 10). A similar energy bias is capitalized on whenever a spacecraft sent from earth toward some other planet is tossed into the gravitational well of an intermediary moving planet to boost or reduce its speed; a maneuver referred to as a *‘slingshot effect’*. By controlling the geometry and timing of the event, the gravitational interaction between the mass of the moving planet and the satellite can be used to advantageously shuffle kinetic energy from one mass into the other.

The energy biases described above are associated with the motion of big lumpy, easily identified objects, but physics does not set any limit on the scale in which such energy maneuvers might occur. Given that one of the unexplained characteristics shared by all organisms on earth is their odd preference for a particular chiral geometry in most key metabolic molecules (proteins, amino acids, DNA, enzymes),^28^ is it reasonable to contemplate whether metabolic process might be able to perform similar energy harvesting maneuvers by manipulating biased actions at the molecular level. One of the immediate obstacles to modeling such conjecture is the significant difference in environments. Satellites traveling in space follow free-fall trajectories governed by the satellite’s momentum and gravitation forces acting to change that momentum. From the Newtonian perspective, the gravitational force is associated with distant masses, while the momentum (the inherited inertial state) is a property that masses carry with them when ‘coasting’ (and not being acted on by external forces). This is very different from thinking about the ‘trajectories’ of objects within the cell constrained by the viscous cytosol.

An insightful article about how biological molecules must manipulate internal strain to generate movement within viscous fluids was written in 1976 by the physicist E.M. Purcell titled: *Life at Low Reynolds Number*.^29^ He points out that in high viscosity environments, motion is very different from that which has fostered our intuition. The ratio of inertial to viscous forces (the Reynolds number) is very low; therefore objects can’t be given a single push like a baseball thrown across a field or a satellite coasting around the planet. Rather objects have to be acted on with coordinated intent along every point of their trajectory, directing them toward their destination. In Purcell’s words: *“inertia* [i.e., momentum] *is totally irrelevant”*. Instead, *“Motion at low Reynolds number is very majestic, slow, and regular.”* Objects control their direction of travel by deforming their bodies in a repetitive, biased way and *“swim”* to destinations. Motion becomes a *learned* process negotiated between body forces releasing internal strain energy and external viscous forces, which work to minimize the impact of the reconfiguration. Notably, the most efficient fluid motions are those which do not break bonding allegiances between neighboring particles (cavitation) but instead are those which allow bonds to deform and oscillate in slightly asymmetrical ways, allowing both object and fluid to wiggle past each other.

**Figure 11.**
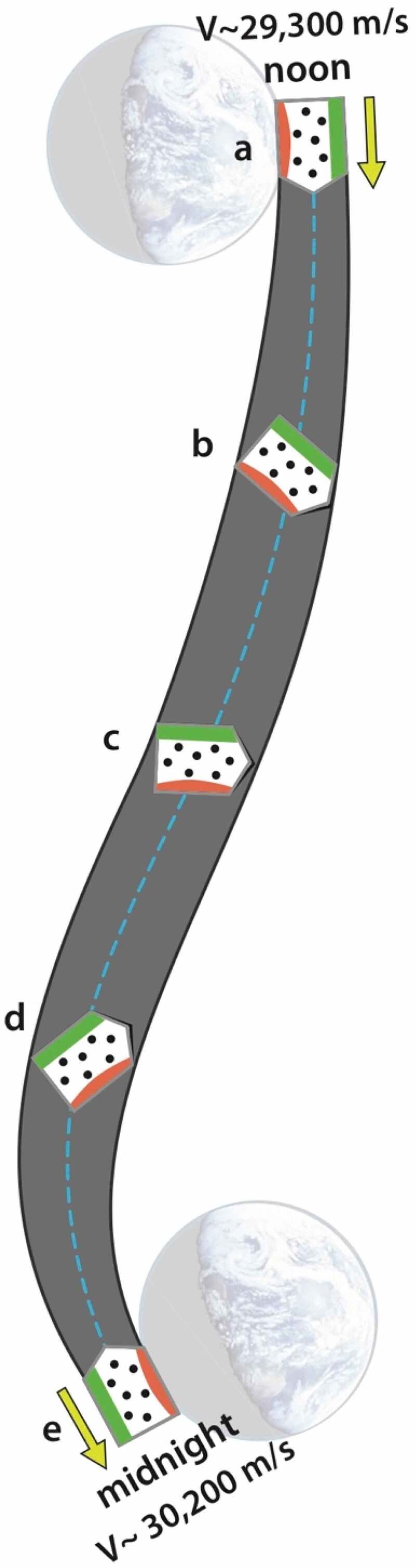
Twisting path of an earthbound object from noon-to-midnight

Purcell’s comments become especially intriguing when juxtaposed with the way external gravitational forces act on organisms wobbling their way along an orbital path. Figure 11 illustrates the orientation of a hypothetical ‘organism’ at five positions a→b→c→d→e over the 12-hours between noon-to-midnight each day. During this time, internal states are accelerated, twisted, and flipped relative to the gravitational frame of the sun (imagined to be at the distant right). Regardless of the smallness of the resulting strain, each of these twists and flips not only resets the local definition of equilibrium (‘up’ and ‘down’), but they also provide an opportunity for the organism to develop patterns of internal motion that become increasing more coherent with the large-scale motion of the system.^30^ This complex state of motion is rich in energy-saving possibilities. If, as Purcell identifies, the greater the coherency between internal motion with the fluid environment binding it, the greater the energy efficiency, then this can serve as evolutionary motivation for organisms to develop proteins, enzymes, and messenger molecules (such as RNA) with configurations that wiggle their internal states in a coherent (or anti-coherent) way with the orbital motion. The state of coherency would likely be expressed deep within the electronic potentials of constituent molecules. Imagine, for example, a chain of bound molecules represented by this sequence of symbols, ⊙−⊙−⊙−⊙−⊙, traversing the path shown in Figure 11. Recall that over 99.9% of the mass of each molecule is located within the nuclei (represented by the inner dots). Thus, three gravitational forces act on those nuclei; their path is guided by the gradients in those gravitational potentials. However, that is not the same path that viscous fluid forces (powered by the rotation and orbital trajectory of the earth) are trying to steer the object; that path is enforced by the electronic structure surrounding those nuclei (represented by the circle around the dots). Shouldn’t we expect the high-energy, high-frequency, proton-electron atomic structure to participate in the overall coherency of the twisting and flipping system – thereby increasing the efficiency of motion even further?

Interestingly, if we compute the distortion in the electron field needed to store the equivalent energy to that of the total daily change in gravitational potential energy in an atom, defined by $\Delta U_{sun}$ in Eq. (1.1), all that is required are coordinated ‘wiggles’ that lead to a mean displacement between proton and electron of roughly one proton diameter. Conveniently, the circadian, the circa-lunar, and the seasonal oscillations in the gravitational potential energy can each be stored within the strain field internal to molecules. If an organism evolved means for coordinating the inward winding and outward release of this strain energy, it could be used to perform metabolic activities. Such small oscillations or deformations would not reveal themselves in the chemical structure because the energy stashed within any single molecule would be too small to cleave a bond. (Cleaving a substrate bond requires the coordinated delivery of gravitational strain from several thousand molecules, such as enzymes are able to accomplish.)^31^

Purcell continues his analysis about propulsion efficiency of spinning helical objects moving through viscous fluids to show how they can be powered by diffusion of energetic molecules in the fluid itself – *the process just has to be coordinated properly*. Importantly, the most efficient movement strategy for such objects is one where a controlled balance exists between the rate of diffusion, the rate of rotation, and the rate of linear progress through the fluid. The dynamics of such motion are counterintuitive, but gravitational forces are not needed to *pull* objects through the cytosol – besides, they are far too weak for that. Rather, the strain pattern generated by gravitation is needed to synchronize the internal movement of a protein with the dynamics of diffusion processes so they can be propelled and directed. Supporting that notion is the observation that orbital strains are precise to within a few milliseconds per year.

Consider how gravitational forces acting on nuclei supported within a fluid must distort the position of those nuclei downwards from the centered positions shown here, ⊙−⊙−⊙−⊙−⊙. This distortion in the electronic field represents an *a priori* condition of all molecules and substances, both organic and inorganic. However, if the substance has a rigid crystal structure (like a piece of granite), there is little opportunity for twisting and folding the large scale (body) structure to provide the benefits that secondary bonding structure offers. Secondary bonding configuration is one of the trademarks of proteins and organic molecules; they provide mechanical connection that allows for coordinating strain in a resonant way throughout the structure. This sets up a fascinating modeling possibility for a weak strain undercurrent where strain energy internal to an organic substance is shuffled within and between molecules in synchronicity with the changing gravitational vectors and the diffusion characteristics of the cytosol. Since organic particles naturally possess the flexibility to twist and wiggle in a coordinated way along our orbital path, rhetorically, wouldn’t their motion resemble Purcell’s swimming organisms?

Finally, a clarification should be made about our preoccupation with referencing motion to the inertial frame of the sun, the Copernican frame. Why not consider energy changes associated with even larger or faster moving frames such as the one related to the velocity of our solar system around the Milky Way galaxy or with the velocity of our galaxy relative to the cosmic microwave background. The justification is that our focus is on systems that undergo significant acceleration cycles within *human* time frames. The velocity of an earthbound organism oscillates in *‘the sun’s frame’* once each day and reverses its direction every six months, while cycles associated with our galaxy’s rotation have periods of over 230 million years.

### The Current Model for Biological Oscillators: the TTFL

The prevailing model used to explain circadian rhythms is based on constructing reliable timing cycles from transcription and translation processes; the model is referred to as the *Transcription-Translation-Feedback-Loop* (TTFL). The intent here is not to debate whether or not the cycles identified by the TTFL exist in the cell; quite certainly they do. But whether this motion is responsible for driving the timing cycle or whether it is itself being driven by some underlying strain mechanism has yet to be determined. The complexity in the TTFL is reminiscent of the complexity of models used to explain planetary motion during the 16^th^ century when epicycles were added to preserve consistency between the observed positions of the planets and the Ptolemaic model. Such complexity, of course, fell away once the Copernican view was adopted.

At a 2019 Conference in Göttingen,^32^ one of the researchers honored with the Nobel prize for work related to the TTFL model pointed to *“three small questions”* that remain unanswered.

1. Why 24-hrs? The cycling period has been observed to take roughly 24-hrs, but there is no specific reason yet identified for the chemical reactions comprising the TTLF cycle to result in 24-hr periodicity. The cell environment is too complex to model numerically, so no computational basis can be provided to justify why the chemically driven sequence should match the earth’s rotational period.
2. Why are circadian rhythms temperature independent? Almost all chemical reactions speed up or slow down with change in temperature; reaction rates usually double with a 10ºC rise in temperature. As noted above, *“whether the organism is living in the bright sunlight of the Mojave Desert where temperatures routinely exceed* 50ºC *or under an overcast Antarctic winter sky at* 20ºC *below zero, or inside the digestive tract of a cow* (39ºC) the organism will experience the same periodicities and patterns of strain.” And yet, the 24-hr periodicity of the TTLF is somehow unaffected?
3. The TTLF does not yet satisfactorily explain how such biological systems maintain their periodicity in dark environments. Whether the organism resides in Longyearbyen, Norway (latitude 77.13 N), which experiences three solid months of sunlight in the summer and three solid months of darkness in the winter, or deep in the aphotic zone where almost no sunlight reaches it, or, per (2) *“inside the digestive tract of a cow”*, the 24-hr periodicity remains intact.

These do not seem like ‘*small’* issues to resolve – Feynman might agree. Further, it has been found that some organisms such as Cyanobacteria express circadian rhythms *without* transcription – the process underlying the TTFL model. This is not to say that transcription, translation, and sunlight do not play any role in circadian cycles; however, perhaps those factors alone do not provide us with the complete picture. In that respect, it may be noted that each of the problems identified above is washed away if organisms can cue, at least partially, to the periodicity in gravitational strain cycles generated by our orbital motion.

## II: the experimental challenge

None of the correlations identified in the first part of this paper are helpful to biologists, zoologists, and botanists unless a mechanism can be envisioned or revealed that demonstrates a causal link. Until such time, they remain a curiosity, *coincidental* at best. However, it may also be argued that while false conjecture only leads to wasted time and null experiments, false skepticism leads to no investigation at all. Recall how, in the early 17th-century, Kepler was fiddling with numbers related to planetary orbits in data obtained from Tycho Brahe when he noticed that the square of the orbital period just happened to be proportional to the cube of the semi-major axis.^33^ Upon finding this pattern, Kepler wrote: *“I first believed I was dreaming … but it is absolutely certain and exact that the ratio which exists between the period times of any two planets is precisely the ratio of the 3/2ths power of the mean distance.”* Kepler’s simple observation became his third law (*Law of Harmonies*) which was critical to Newton when he later derived his gravitational equations. The challenge for biologists is to imagine how biological processes might have evolved to take advantage of the gravitational potential energy undercurrent, then devise experiments that can test such conjecture. Some of the experimental difficulties are identified below, followed by an outline of experiments that *might* generate valuable results.

### Gravitational strain amplitudes are below detection thresholds

It was shown in Figure 6 how the acceleration of an organism, or an object located on the surface of the earth, is roughly $5900\mu /s^{2}$ toward the sun, and $32\mu /s^{2}$ toward the moon. Those accelerations are a simple consequence of Newton’s formula $F=-GMm/R^{2}$ applied to the instantaneous orbital configuration. However, these accelerations are not easily sensed within a cell since the entire fluid environment is experiencing similar acceleration. Generally, mechanical systems sense *relative* accelerations which are then associated with tidal forces. Tidal accelerations are approximated by the tidal relationship $\Delta g_{tidal}\approx \nabla g\cdot \Delta r\approx 2GM/R^{3}\cdot \Delta r$ and are many orders of magnitude smaller.^34^ Tidal effects from the sun and moon acting on the earth are caused by a difference in relative acceleration of $\Delta g/g10^{-7}$. But, if we apply the same relationship to particles separated by cellular dimensions, say 10 microns or so, the tidal accelerations due to the sun and moon are roughly $3\times 10^{-18}m/s^{2}$ and $2\times 10^{-16}m/s^{2}$. Therein lies the problem: tidal accelerations are extremely small, so it is easy to simply adopt the position that they don’t matter and move on.^35^

### Thermal noise

Explaining how a weak gravitational coupling might be maintained within a noisy thermal environment is a challenge. Even more difficult is the practical aspect of observing the means amidst a sea of Brownian motion. However, these seemingly intractable problems become a little less so with closer scrutiny of the nature of the gravitational coupling. A preliminary analogy can be made with the phenomena of lightning. Lightning is an electrical discharge accompanied by two phenomena, a flash of light and a rumble of thunder. One is an effect propagated at the speed of light, the other at the speed of sound; it is not difficult to tell them apart. In a similar way, whether we adopt the Newtonian perspective that gravitational forces act instantaneously over distance, or the Einsteinian view that imagines gravity as a local curvature of spacetime where changes are propagated at the speed of light, there is a significant difference between the resulting gravitational and thermal vibrations. The power spectrum of thermal noise and in Brownian motion falls off exponentially at very high frequencies. The upper limit for sound vibrations within a molecular lattice is identified with phonons, estimated to be $10^{15}Hz.$ If gravitational strains are curled-up within the electronic field internal to atomic structure, their interaction frequency is likely closer to the Bohr frequency, which is another order of magnitude higher. Signals in that domain may fall into a relative ‘quiet zone’ with respect to thermal noise, but they will be a challenge to identify. Advice might be obtained from astronomers who are able to extract small wobbly periodic cycles in the light spectrum from distant stars indicative of an exoplanet.

Scientists have given the ISS the label of being experimentally ‘noisy’ due to vibrations from pumps, fans, thrusters, and such. However, the highest frequency noise on the ISS seems to be generated by a Russian Carbon Dioxide Removal System (*Vozdukh*),^36^ which has a frequency of ~12,500 Hz. For reference, this is twice the frequency of a jet engine, but is unlikely to interfere with the gravitational coupling if such coupling operates in the range of nuclear or terahertz frequencies.

### Light entrainment in circadian experiments

If strain from orbital periodicity does cue metabolic oscillations, then there is a need to explain results obtained from many, many experiments in the field of circadian rhythm research that demonstrate how metabolic cycles can be (at least partially) entrained to the periodicity of artificial light cycles [9]. This solution to this puzzle may also fall into the abyss that currently exists in our understanding of how gravity is to be integrated with quantum mechanics. Photosynthesis is well understood at biological scales, but it is unclear what the photon absorption process might have on the gravitation coupling. If biological molecules are able to slowly wind, or curl-up, weak tidal strains into their electronic states, then they likely do so within the energy limits established by their chemical bonds. Periodicity in such a subtle process would likely be shattered by the absorption of high-energy photons that reset the electron configuration. Since even the lowest levels of light used in circadian experiments send a flux of trillions of photons per second through a system, it likely that coherency in the gravitational strain current is temporarily overridden. This might be investigated by monitoring photosynthesis rates with high-resolution under different strain conditions (both artificial and natural).

### Microgravity simulation devices

There is a century-long history of experiments utilizing clinostats, random positioning machines, rotating wall vessels, and other *‘microgravity simulation devices’* to measure how plants and organisms respond to altered inertial environments [10–12]. A misconception arises from the use of the term ‘microgravity’ and the implication that these devices alter the gravitational field. Microgravity simulation devices *add* artificial convective forces and shear stress to fluids in contact with bio-samples in a manner which counters the downward acceleration they would otherwise experience due to gravity.^37^ A 2019 paper by longtime researchers in the field, Kiss, Wolverton, Wyatt, Hasenstein, and van Loon [13] titled, *Comparison of Microgravity Analogs to Spaceflight in Studies of Plant Growth and Development*, summarizes this misconception. First, they remind us that the trajectory of objects in orbit is the result of *“balance between centripetal force of gravity and the centrifugal force of the moving object … This balance is often mistakenly referred to as microgravity, but is best described as weightlessness.”* And that gravity simulation devices *“do not reduce gravity but constantly change its direction.”* Their conclusion:

*“Numerous studies have compared the biological effects of clinostats and other microgravity analogs to space experiments. The experiments to date suggest that while these devices may be useful tools in some cases, there are great differences observed between plants that grow and develop on these devices and plants that are grown in weightlessness during spaceflight.”*^38^

Such experiments do provide useful insight on the *bio-inertial* response; however, they can misrepresent the bio-gravity response and need to be used with caution.

### Drop Towers, Parabolic Flights, and Mountain Tops

Assuming the gravitational coupling cycles strain over 24 hours, or 29.5 days periods, then experiments performed in drop towers (duration ~10 seconds) or during parabolic flights (duration ~ 30 sec) are not likely to reveal the coupling. However, if the coupling cycles with the gravitational potential, $\Delta U_{\left\{e,m,s\right\}}$, then differences might be observed in metabolic indicators such as gene expression or ATP production rates when identical biological systems in identically controlled growing environments are placed in different gravitational potentials and observed over long time periods. Two organisms – one placed atop Mauna Kea in Hawaii (4,200 meters above sea level) and the other deep in a South African gold mine (3,200 meters below sea level), for example, will experience a difference of $2.3\times 10^{-2}m/s^{2}$ difference in earth’s gravitational acceleration. This is just slightly less than the $3.0\times 10^{-2}m/s^{2}$ acceleration sensitivity presently detectable in gravitropism experiments. More revealing but more challenging would be finding means for detecting the ‘noon-to-midnight’ cycle in $\Delta U_{sun}$ and $\Delta U_{moon}$ associated with seasonal variations; roughly $5.8\times 10^{-10}m/s^{2}$ and $1.3\times 10^{-9}m/s^{2}$, respectively.^39^

### Alternative Orbits – (Where No Organism Has Gone Before)

Most of the recent direct data gathered about the effects of spaceflight on organisms has been obtained from ISS experiments. While ISS results are extremely valuable, they present a problem when trying to identify more specific information related to the possible biogravity coupling because the ISS orbit is fixed. The trajectory of organisms aboard the ISS, relative to the sun’s gravitational potential is, therefore, very similar from orbit to orbit, and from day to day. For example, over the 8 days in which the *Arabidopsis* plants shown in Figure 2 were grown, they were made to oscillate back and forth within both the sun’s and moon’s gravitational potential roughly 125 times, but at a substantially fixed inclination every 93 minutes.

If similar future experiments were to be performed aboard spacecraft traveling in orbits with different geometries or periods, it might be possible to separate out the gravitational contributions from the sun and moon in the data. A polar orbit during the fall or spring equinox periods oriented in the plane perpendicular to the radial vector to the sun, for example, would remove most of the oscillation of the sun’s gravitational potential from the orbital path. Similarly, a plant placed in a higher or a geosynchronous orbit would experience a longer tidal cycle and different $\Delta U_{sun}$ and $\Delta U_{moon}$ properties. The most revealing experiment would entail sending a spectrum of plants and other organisms out toward some distant planet (mars?) and monitor their metabolic characteristics relative to equivalents groups both earthbound and aboard the ISS-bound.^40^

There is one other difference between strain cycles experienced by earthbound organisms and those in orbit worth noting. Both the fluid and solid regions of the earth undergo tidal distortions from the sun and the moon. As the surface supporting an organism swells beneath it (whether in the ocean or on land), the gravitational and centrifugal vectors wobble in divergent ways.^41^ The effect is small, of order $\approx 10^{-13}$, but it is absent from organisms in free-falling orbits.

### ‘in-silico’ experiments

*“Computer models aim to incorporate multi-scale biological data into comprehensive, dynamic computer software that simulates a biological system.”*^42^

A perspective has been growing that computer simulation experiments, playfully dubbed *‘in-silico’* experiments, will be able to mimic *in-vitro* and *in-vivo* experiments. Maybe that will be true for some biological processes; however, currently, gravitational interactions are excluded from all input data. Thus, gravitational effects will never show up in their output. Further, even the most powerful computing devices used today can only return simulations spanning a few seconds in duration – way too brief to reveal effects contributing to circadian or circalunar events. This omission of gravity from the computer modeling has repercussions that extend beyond the obvious domains where we think gravity might have influence. For example, research has shown that maintaining synchronicity is critical for a properly functioning immune system, and when that order is perturbed, it can cause serious metabolic effects, including, possibly cancer [14]. If gravitational strain cycles assist in regulating metabolic synchronicity, that dependency will never be exposed in currents simulations. Where computer simulations would be valuable is in helping model how complex shapes such as proteins and enzymes might harvest energy from strain gradients set up between their boundary couplings and gravitationally driven accelerations acting on their interior.

### Modern Gravitational Experiments

Modern physics provides several fascinating theoretical avenues for exploring the biogravity coupling. Special relativistic effects related to daily and seasonal velocity changes produce an oscillation in the gamma factor $\gamma \left(t\right)={\left(1-{\left(v\left(t\right)\right)}^{2}/c^{2}\right)}^{-1/2}$associated with mass, length and proper time. Due to the velocity changes experienced by organisms rotating and orbiting with the earth (see Figure 6), the proper units of mass, length, and time oscillate by a factor of roughly $3\times 10^{-10}$ over the course of each day and by roughly the same amount with the changing seasons. Such oscillations are shared by the co-moving frame of a cell, but the cycle might be detectable if referenced to the rotating frame of the earth or the light spectrum from larger inertial frames.

Investigating for possible cycles in the Casimir effect might also give insight into the gravitational coupling. The Casimir effect is a force acting on particles generated by quantum fluctuations in the vacuum energy. While they were originally associated with large planar shapes, they are now expected to act between particles of all shapes both at biological scales and at scales that contribute toward chiral order in nuclear structures.^43^ If the strength of the Casimir force is coupled to the gravitational potential, then it would cycle within an organism as the position of the organism (rotating with the earth) cycles relative to the sun – moving deeper into the sun’s gravitational field during the day and further away at night. Whether such cycling would produce detectable changes in the performance of proteins and enzymes is uncertain.

Interestingly, the notion that our innermost clocks might oscillate in phase with changes in the gravitational potential is consistent with our understanding of gravitation redshifts. Oscillation frequencies slow down when moving deeper in a gravitational field and they speed up when moving higher. The shift in frequency experienced by an organism as its position swings back and forth within the sun’s field (between noon and midnight) is only of order $\approx 10^{-12}$, but it is hard to know when small effects warrant dismissal.^44^ Difficulty in detecting such as shift is increased because the shift has to be referenced to larger boundary conditions of their inertial environment. On the encouraging side for experimentalists is that gravity wave detectors have reached a sensitivity of one part in $10^{22}$.

### Biological improvisation

Not to be forgotten is the reexamination of physical theories that have been applied to biology because they seemed proper at the time, but which were formulated based on observations of inanimate matter and might not apply in biological domains. Perhaps the best example of this was pulled off by biologists when they modeled how substrates were able to circumvent thermodynamic limits and speed up conformational changes by partnering with enzymes. This quote by biophysicist Ehud Gazit summarizes the extent to which previous rules were overridden^45^

The biochemical reactions taking place in cells, though thermodynamically favorable, are in most cases very slow if left uncatalyzed. Fortunately, metabolism is carried out by enzymes that often increase rates by an astonishing 10 orders of magnitude or more. Reactions necessary to create hemoglobin would take 2.3 billion years … enzymes reduce this time to mere milliseconds.

If there was ever a cue for gravity to enter the discussion, that was it – *“Increase rates by 10 orders of magnitude”* – that is just what is needed to bring gravitational forces the attention they need. Perhaps biologists can perform a similar improvisation and find theoretical means to understand how weak gravitational strains might be similarly magnified.

## Final remarks

In 1940 Linus Pauling began his comprehensive treatise *General Chemistry* by reminding students that *‘the liberated energy’* in all chemical reactions comes from a reduction in mass defined by the relativistic equation E = mc^2^. Pauling then calculated the change in mass for a typical high-energy chemical reaction to illustrate how the 1 part in 10^10^ mass loss tends to fall off the chemist’s radar, but that the critical connection between chemistry, mass, and fundamental physics, in general, should not be forgotten. That reminder applies to all research fields contributing to biological modeling today, for biologists have extended their level of investigation into scales where not only does the definition of mass come into scrutiny, but also most other fundamental assertions in physics.

Scientists from many eras have held the belief that gravitational forces might be playing a role in biology because organisms on earth seem to share a dynamic order, a periodicity, and a handedness in both their structure and their interaction that is hard to understand without appealing to some common means for choreographing these qualities. Organisms have evolved multiple chemical energy conversion cycles to power their metabolisms; thus, they may not need the internal strain generated by gravitation anymore as a source of energy. However, since gravity has the unique distinction of being the only force in nature that acts on all matter on earth in a common, familiar way, it seems feasible gravitational cycles are still essential in regulating coherence within core metabolic functions. As space scientists prepare to send organisms out away from the inertial environment in which they evolved, and as advances in medical research seek to understand metabolic order at increasingly finer scales, it is critical we assess whether or not gravitational strain cycles do participate in synchronizing life’s order. If Pauling were still alive today, he would likely be reminding students to keep gravitation forces on their modeling palette for that very reason. Energy released in chemistry bonds may indeed come from the conversion of mass into energy; however, that energy is raw, immature, and stochastic – something has to act as a conductor or chaperone to convert that energy into a bias functional form useable to a cell. Excitingly now, both the opportunity and the motivation exist within numerous research fields to finally put the full Copernican perspective about gravity to experimental inquiry.

## Notes

^1^ For example, the word ‘gravity’ cannot be found in major textbooks such as *Biochemistry* by Stryer, Berg, & Tymoczko, 2002, W.H. Freeman and Co.; or, *Introduction to Genetic Analysis*, [publisher]; or, *The Molecular Biology of the Cell*; or, *Gene*, by Siddhartha Mukherjee. The subject of gravity was almost omitted from the 2018 Biophysical Society conference in San Francisco where there were over 7600 attendees.

^2^ See the published summary of the conference lectures: *Gravity and the Organism*, Edited by Solon A. Gordon and Melvin J. Cohen, University of Chicago Press, 1971.

^3^ The effect of spaceflight on the gravity-sensing auxin gradient of roots. Robert J Ferl and Anna-Lisa Paul *npj Microgravity (2016)* 2, 15,023; doi:10.1038/npjmgrav.2015.23

^4^ November 2020 issue of Cell Press.

^5^ For an article describing the effects of microgravity on microbes, see Huang et al. Effects of spaceflight and simulated microgravity on microbial growth and secondary metabolism. *Military Medical Research* (2018) 5:18 https://doi.org/10.1186/s40779-018-0162-9

^6^ George Freier and F. J. Anderson.

^7^ Bernard Schutz, Gravity from the Ground Up (2003) Cambridge University Press.

^8^ There is no agreed upon formula for calculating BMR, which is why a range is provided. We can get a sense of the value by recalling how most dietary guides suggest a calorie intake of ~ 2000 kcals per day for a typical active adult. BMR is less because it reflects the minimum energy needed to sustain the individual when they are at rest.

^9^ Makarieva et al. PNAS, November 4, 2008, vol. 105, no. 44.

^10^ Ibid, footnote 4.

^11^ *“Plants have evolved under the influence of Earth’s gravity, a force of “1 g.”* Kiss JZ, Wolverton C, Wyatt SE, Hasenstein KH and van Loon JJWA (2019) Comparison of Microgravity Analogs to Spaceflight in Studies of Plant Growth and Development. *Front. Plant Sci. 10:1577*. doi: 10.3389/fpls.2019.01577

^12^ The extremely accurate periodicity of our orbital motion is what allows astronomers to predict solar eclipse events one thousand years in advance. See, for example, NASA’s Five Millennium Catalog of Solar Eclipses: https://eclipse.gsfc.nasa.gov/SEcat5/SE2901-3000.html

^13^ Figure 6 is also a simplified view of the geometry; it does not include the tilt of the earth’s axis or of the moon’s orbit, both of which add cyclical variation to the system.

^14^ There is a reduction in the effective radius due to the $\left(23.5^{\circ }\right)$ tilt of the earth’s axis relative to our orbital plane; this important reduction alters the values used here by ≈7%.

^15^ If we average the energy change over the full circadian period of 24-hrs, the mass-specific-power drops in half to ${\hat{P}}_{n\to m}=0.87Watts/kg$ – still within the lower range shown in (Figure.4).

^16^ More precisely, if we include the tilt of the earth $\begin{array}{l} \Delta {\text{U}}_{\text{sun}}={\text{m}}_{\text{ATP}}\cdot \frac{\text{G}\cdot {\text{M}}_{\text{sun}}}{{\text{R}}_{\text{sun}}^{2}}\cdot 2{\text{r}}_{\text{earth}}\\ \cdot \text{Cos}(23.5{}^{\circ})=36\text{kJ}/\text{mole} \end{array}$

^17^ See *‘daily turnover rate of ATP’* at bionumbers.hms.harvard.edu/search.aspx?trm = daily+turnover+rate+of+ATP

^18^ *Annual, Lunar, and Tidal Clocks* Editors: Numata, Hideharu, Helm, Barbara, 2014 Springer.

^19^ Since BMR and ATP energies were tied to the change in potential energy relative to the sun, then we might also expect to find seasonal cycles in their values. There should also be a correlation with latitude since the effective daily shift in distance relative to the sun varies proportionally to $r_{e}Cos\left(\theta \right)$. Correlation of metabolic rates with latitude are observed in many species and can be seen most generally in how the robustness of life falls off with distance from the equator.

^20^ The difference between seasonal centrifugal and gravitational accelerations, defined here by $\alpha =\left(a_{o}-g_{s}\right)$, can be calculated simply by inserting the value of earth’s position and velocity into the acceleration formulas, $a\left(t\right)=V{\left(t\right)}^{2}/R\left(t\right)$ and $g_{s}\left(t\right)=GM_{s}/R{\left(t\right)}^{2}$.

^21^ In 2017, the author asked the speaker kicking off the conference on Biological Oscillators at the EMBL Heidelberg (who had been describing the periodicity their group detected in the gene expression in a midge while standing in front of a huge screen image of the earth, the sun and the moon, much like one would see in an astronomy lecture), *“Have you considered gravitational strain as a possible coupling mechanism?”* Her reply: *“No, why would we consider that?”*

^22^ See the discussion in Part II about the possibility of the photon flux overriding the gravitational signal.

^23^ Levi et al. Light-Responsive Cryptochromes from a Simple Multicellular Animal, the Coral *Acropora millepora Science* Vol. 318 19 OCTOBER 2007.

^24^ ISS orbit decays slightly due to atmospheric drag and has to be re-boosted back into position each month.

^25^ This is really just another way to state that our motion results from the interaction of all three gravitating bodies acting on it, but by acknowledging the wobble in advance, we prevent the interaction from being truncated out of the conversation.

^26^ Fisahn experiment reference. *Annals of Botany* 110: 301–318, 2012. doi:10.1093/aob/mcs038. Available online at www.aob.oxfordjournals.org

^27^ At least one member of this research team is now engaged in an earthbound experiment which will subject similar plants to artificial accelerations of similar magnitude to that produced by the gravitational fields of sun and moon but with an opposite phase, therefore seeking to observe the removal of known motions that the plants would otherwise produce. (Private correspondence).

^28^ *Chirality in Biology* Wiley Online Library Supplement 1. Biochemistry Ronald Bentley 15 September 2006https://doi.org/10.1002/3527600906.mcb.200200008

^29^ American Journal of Physics vol 45, pages 3–11, 1977.

^30^ Such opportunity is not availed to any system described mathematically as a point.

^31^ This resembles the enzymes-substrate interaction; see the quote in Part II.

^32^ 2019 *Horizons in Molecular Biology Conference* at the Max Planck Institute for Biophysical Chemistry in Göttingen.

^33^ The ratio $R^{3}/T^{2}$ for Mercury, Venus, Earth, Mars, Jupiter and Saturn were all ~ 7.5for each of the planets He observed that the coefficients {7.64; 7.52; 7.50; 7.50; 7.49; 7.43} in the ratio $R^{3}/T^{2}$.//expressed in units fundamental to orbits $\left[AU^{3}/day^{2}\right],$each seemed to express the same ratio $7.5\times 10^{-6}$. Modern values for the coefficient in the ratio $R^{3}/T^{2}$ of Mercury, Venus, Earth, Mars, Jupiter and Saturn are, respectively, {7.496; 7.496; 7.496; 7.495; 7.504; 7.498}.

^34^ See *Fundamentals of Geophysics*, Second Edition. William Lowrie. 2007. Cambridge University Press.

^35^ Measuring acceleration changes relative to the sun may be analogized with trying to sense the status of one’s wealth on a global scale but forced to do so only by comparing the quality of one’s house or car relative to others in the neighborhood. If the value of the currency used by the country changes, that will likely be critical to the health of the neighborhood, but the absolute wealth will be hard to discern because the wealth of the neighborhood will rise and fall together.

^36^ See the NASA./American Institute of Aeronautics and Astronautics report: International Space Station Acoustics – A Status Report, by Christopher S. Allen & Samuel A. Denham.

^37^ Ibid, see Footnote 37.

^38^ See also these quotes from Herranz et al. Ground-Based Facilities for Simulation of Microgravity: Organism-Specific Recommendations for Their Use, and Recommended Terminology. *Astrobiology* Volume 13, Number 1, 2013 *“Simulator experiments have provided excellent insight into a multitude of gravity-dependent phenomena. However, many researchers failed to compare results from experiments in the ‘‘real’’ microgravity of a spaceflight mission with experiments on simulators, with respect to the sample used and to the parameters investigated.” “Without this direct comparison, it is difficult to conclude whether biological reactions or organismic re sponses are caused by the conditions of simulated micro- gravity or by any of the possible side effects of the simulation technique.” “Each type of simulator has its specific artifacts”.*

^39^ The highest resolution of such/ref: an accelerometer is 10^−12^ m/s^2^, and its main uses involve fundamental physics, such as the measurement of earth’s gravity field and tests of the equivalence principle.

^40^ The period of rotation of mars is very close to that of the earth (24.6 hours), but there is a 7-month journey to get there when organisms will face inertial silence. Results from such experiments would give supporting insight into whether plants and organisms can be grown in the altered strain environments of planned moon or mars bases.

^41^ Ibid, see the geodynamics book referenced in note 41.

^42^ Cell studio: A platform for interactive, 3D graphical simulation of immunological processes. APL Bioeng. 2, 026107 (2018); https://doi.org/10.1063/1.5039473

^43^ The Casmir effect is a force acting on particles generated by quantum fluctuations in the vacuum energy expectation level. It also plays a role when modeling chiral effects in nuclear structure. See Griffiths, D. J.; Ho, E. (2001). For the effect on non-planar shapes, see: Classical Casimir effect for beads on a string. *American Journal of Physics*. 69 (11): 1173. Bibcode:2001AmJPh.69.1173 G . doi:10.1119/1.1396620.

^44^ In a gravitational field $\Delta \lambda /\lambda \approx g\cdot \Delta R/c^{2}$; for g(sun) and distance shifts equal to the earth’s diameter this equates to $\approx 8.4\times 10^{-13}$

^45^ Quote from biophysicist Ehud Gazit from Tel-Aviv University.

## References

[1] Ferl RJ, Paul A-L. The effect of spaceflight on the gravity-sensing auxin gradient of roots: GFP reporter gene microscopy on orbit. Npj Microgravity. 2016;2(1):15023. 28725721 10.1038/npjmgrav.2015.23 PMC5515520

[2] Huang, Huang B, Li D-G, et al. Effects of spaceflight and simulated microgravity on microbial growth and secondary metabolism. Mil Med Res. 2018;5(1):18. 29807538 10.1186/s40779-018-0162-9 PMC5971428

[3] Kiss JZ, Wolverton C, Wyatt SE, et al. Comparison of microgravity analogs to spaceflight in studies of plant growth and development. Frontiers in Plant Science. 2019;10:1577. 31867033 10.3389/fpls.2019.01577 PMC6908503

[4] Raible F, Takekata H, Tessmar- Raible K. An Overview of Monthly Rhythms and Clocks. Front Neurol. 2017;8:189. 28553258 10.3389/fneur.2017.00189 PMC5428424

[5] Numata H, Helm B, editors. Annual, Lunar, and Tidal Clocks. Heidelberg: Springer; 2014.

[6] Biscontin A, Martini P, Costa R, et al. Analysis of the circadian transcriptome of the Antarctic krill Euphausia superba. Sci Rep. 2019;9(1):13894. 31554872 10.1038/s41598-019-50282-1 PMC6761102

[7] Kaiser, Kaiser TS, Poehn B, et al. The genomic basis of circadian and circalunar timing adaptations in a midge. Nature. 2016;540(7631):69–73. December 01. 27871090 10.1038/nature20151 PMC5133387

[8] Barlow PW, Fisahn J. Lunisolar tidal force and the growth of plant roots, and some other of its effects on plant movements. Ann Bot. 2012 Jul;110(2):301–318. 22437666 10.1093/aob/mcs038 PMC3394636

[9] LeGates TA, Fernandez DC, Hattar S. Light as a central modulator of circadian rhythms, sleep and affect review. Nat Rev Neurosci. July 2014; 15 (7): 443–454. PMID 24917305 24917305 10.1038/nrn3743 PMC4254760

[10] Borst AG, Jack J, Van Loon WA. Technology and developments for the Random Positioning Machine, RPM. Microgravity Sci Technol. 2009;21(4):287–292.

[11] Dedolph RR, Dipert MH. The physical basis of gravity stimulus nullification by clinostat rotation. Plant Physiol. 1971;47(6):756–764. 16657700 10.1104/pp.47.6.756 PMC396766

[12] Wuest SL, Stern P, Casartelli E, et al. Fluid dynamics appearing during simulated microgravity using random positioning machines. PLoS ONE. 2017;12 1:e0170826.pone.0170826 28135286 10.1371/journal.pone.0170826 PMC5279744

[13] Kiss JZ, Wolverton C, Wyatt SE, et al. Comparison of microgravity analogs to spaceflight in studies of plant growth and development. Front Plant Sci. 2019;10:1577. 31867033 10.3389/fpls.2019.01577 PMC6908503

[14] Scheiermann C, Gibbs J, Ince L, et al. Clocking in to Immunity. *Nat Rev Immunol* 18 July . 2018. 10.1038/s41577-018-0008-4 29662121

